# Supplementary material for: Rare variant phasing and haplotypic expression from RNA sequencing with phASER
Source: Nat Commun. 2016 Sep 8;7:12817. doi: 10.1038/ncomms12817 (PMC5025529; doi:10.1038/ncomms12817)
Supplement: Supplementary Information — Supplementary Figures 1-7 [file ncomms12817-s1.pdf]

Supplementary Information

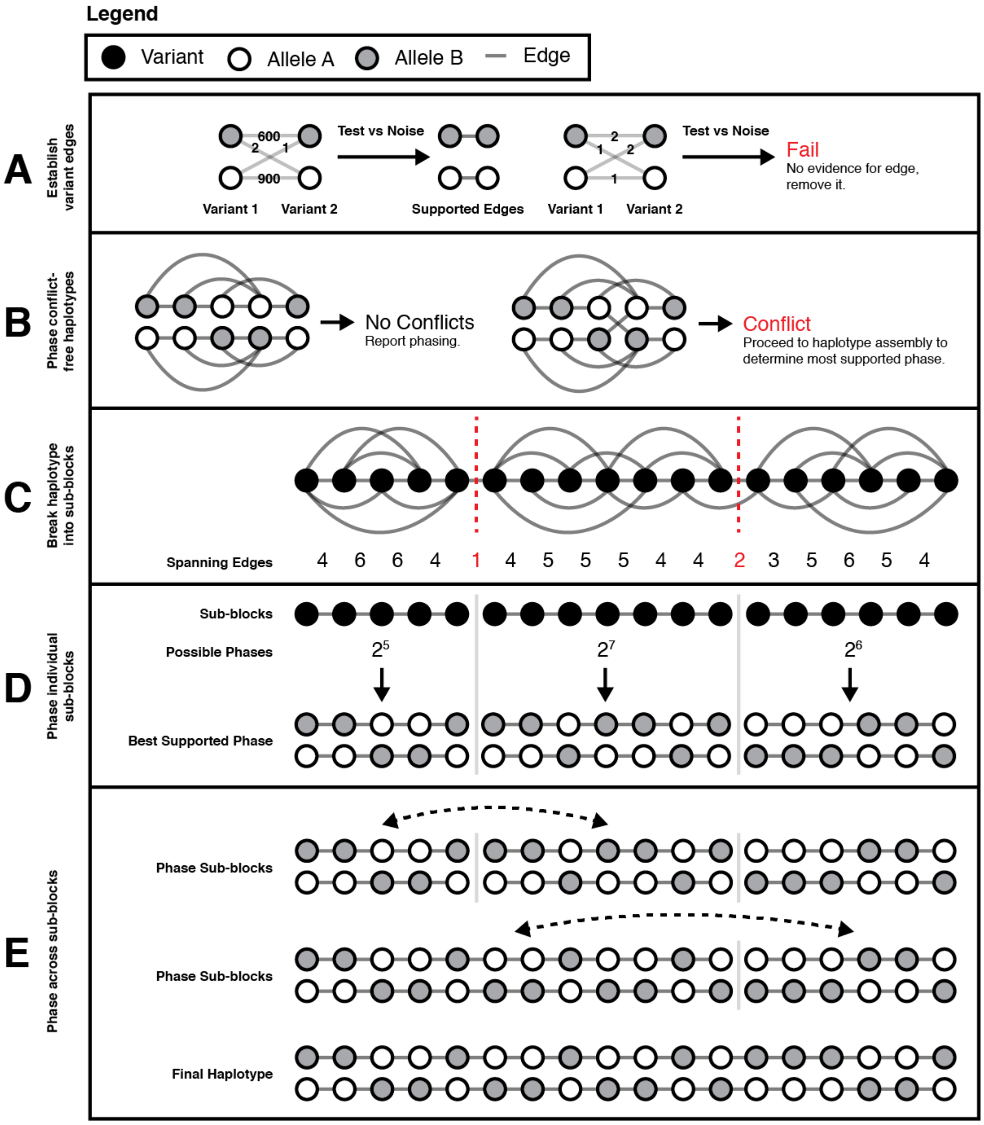

Supplementary Figure 1. Haplotype assembly in phASER. A) The edges between alleles of each pair of variants that are connected by at least one read are defined by selecting the phase configuration with the most supporting reads. A binomial test is performed to determine if the number of reads supporting alternative phase configurations can be explained by the amount of sequencing noise in the experiment (Supplementary Fig. 2a). Any variant pairs that fail this test have all edges removed for subsequent haplotype assembly. B) Using the allele edges defined in (A) individual haplotypes are assembled by starting with a single unphased allele and recursively adding all other connected alleles. If after this process two distinct haplotypes arise, where each haplotype contains only a single allele of each variant, the phase is determined to be conflict free and immediately reported. In cases where a single phase is not resolved, haplotype assembly is used to determine the phase most supported by sequencing reads. C) Haplotypes are split into sub-blocks such that they contain no more than a user-defined number of variants per block (shown here for a maximum of 7 per block). Haplotypes are split at positions with the fewest number of edges spanning them. D) Within each sub-block the number of allele edges that support each possible haplotype configuration is tested, and the most supported phase is selected. The number of possible configurations is equal to 2^number of variants. E) Once sub-blocks have been internally phased, they are then phased relative to one another, again selecting the configuration with the most support, starting by phasing the two most upstream sub-blocks with each other, and then subsequently phasing each downstream sub-block until a single haplotype phase is obtained.

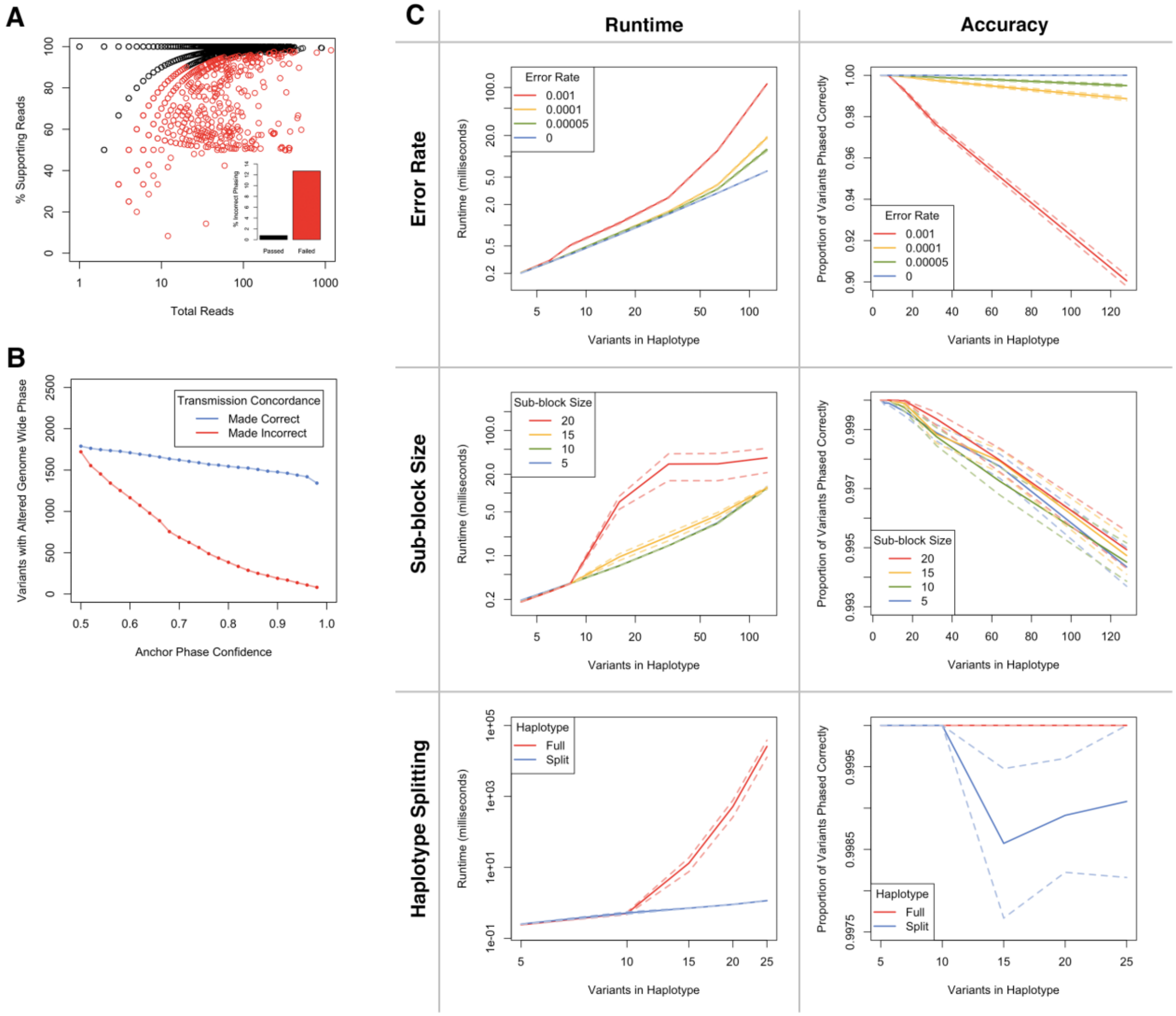

Supplementary Figure 2. Benchmarking of haplotype assembly and phase anchoring in phASER. A) Percentage of reads supporting the chosen phase as a function of total reads at each variant – variant connection for phASER run using NA12878 RNA [11] + WES data [15]. Each point represents a variant – variant phasing, points in black passed the phase confidence test, while those in red failed (evidence for a conflicting phase configuration, $p < 0.01$). See "Statistical test for conflicting phasing between two variants" in the methods section for more detail. The inset bar plot shows the percentage of variant pairs phased incorrectly (versus transmission phasing) for variant connections that passed the test (black) and those that failed (red). B) The anchor confidence statistic robustly removes incorrect genome wide phasing as shown by the number of variants with genome wide phase either made correct or made incorrect as a function of anchor phase confidence with phasing by transmission as a ground truth for NA12878 run with 1000 Genomes exome [15], Illumina Platinum Genomes WGS (5x) and LCL RNA-seq data [11]. See "Genome wide phasing using phase anchoring" in the methods section for more detail. C) Benchmarking of the runtime and accuracy of haplotype assembly used in phASER. Observed variant connections within haplotypes of increasing size (defined by number of variants) were simulated 1000 times using defined error rates (probability of edges between the alleles of two variants being incorrect given a known true phase), and with the same distribution of connections across variants within a haplotype as observed in WGS. For reference, the observed error rate of the NA12878 LCL RNA-seq library was calculated as 6.77e-5, so 5e-5 is included as a comparison. Haplotype assembly was then performed using the simulated haplotypes and variants edges with the phASER method, and both runtime and accuracy were reported. This was done while holding sub-block size constant at 10 while varying the error rate, holding error rate constant at 5e-5 and varying sub-block size, and either phasing splitting haplotypes into sub-blocks of 10 variants at most, or without splitting.

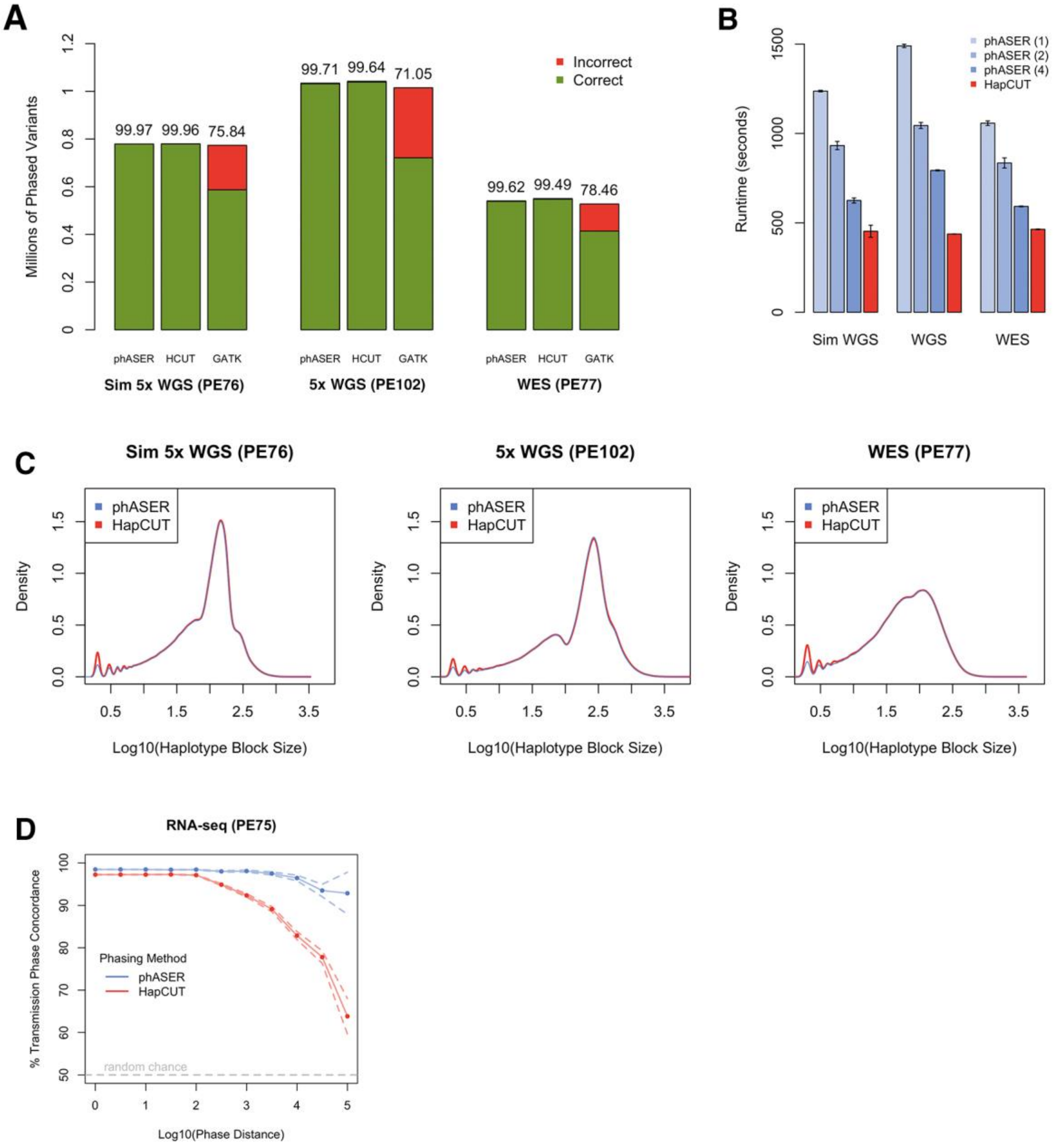

Supplementary Figure 3. Variant phasing is efficient and accurate using phASER. Comparison of phASER to HapCUT and the GATK Read Backed Phasing tool, using either simulated 5x WGS (NA06986, PE76), experimental 5x WGS (NA12878 Illumina Platinum Genome, PE102), or WES (NA12878 1000 Genomes phase 3 [15], PE77). phASER and HapCUT are able to phase a similar numbers of variants, at high accuracy (A), with comparable runtimes (B), and identical haplotype block sizes (C). Runtime for phASER is shown using 1, 2 or 4 threads, since parallelization is available, unlike in HapCUT. phASER's increased runtime is a result of phASER being designed from the ground up to work with RNA-seq reads, including many features such as additional quality control (QC) and allelic expression reporting that are necessary when using this data type, even though they increase overall runtime. Runtime values are means across 4 replicates, and error bars show the standard error of the mean. In our tests, the accuracy of the GATK tool was poor, so it is not shown in subsequent benchmarks. Accuracy was determined by comparing the inferred phase to the known true phase (either the haplotypes used to simulate reads, or the transmission phased NA12878 haplotypes). D) Comparison of phasing accuracy vs distance for phASER and HapCUT when used with PE75 RNA-seq from NA12878. While HapCUT will run using RNA-seq data it lacks the additional QC features found in phASER that are designed specifically to accommodate for the increased alignment error rate in spliced RNA-seq reads. This results in vastly decreased performance at large distances when compared to phASER.

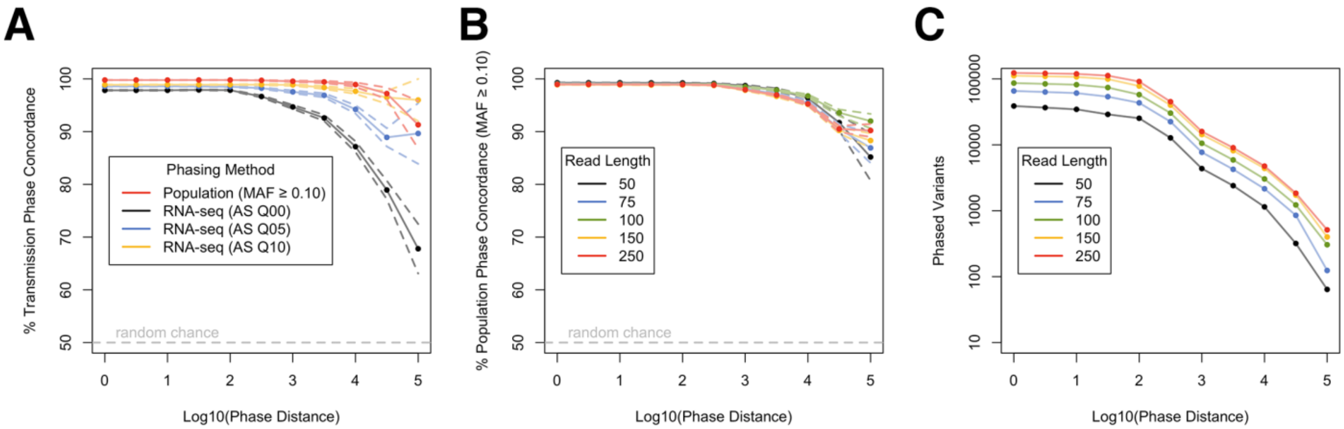

Supplementary Figure 4. Effects of alignment quality and read length on RNA-seq read backed phasing. A) Comparison of RNA-seq phasing using either no alignment score cutoff (AS Q00), or cutoffs equal to the bottom 5% (AS Q05), and 10% (AS Q10), and population based phasing of common variants (MAF ≥ 0.10) to phasing by transmission. For common variants the accuracy of population phasing is not expected to decrease with distance, so it is shown here as a comparison. Generated using NA12878 LCL RNA-seq data [11], 1000 Genomes Phase 3 population phasing [15], and Illumina Platinum Genome transmission phasing. B-C) Comparison of RNA-seq phasing to population phasing for variants with MAF > 10% at increasing read lengths (B), and number of variants phased at equal to or greater than increasing genomic distances for increasing read lengths (C), using a GTEx [12] long read RNA-seq library (GTEX-WFON-0001-SM-5S2SE) clipped to various lengths. Solid lines represent the means, and dotted lines the standard error.

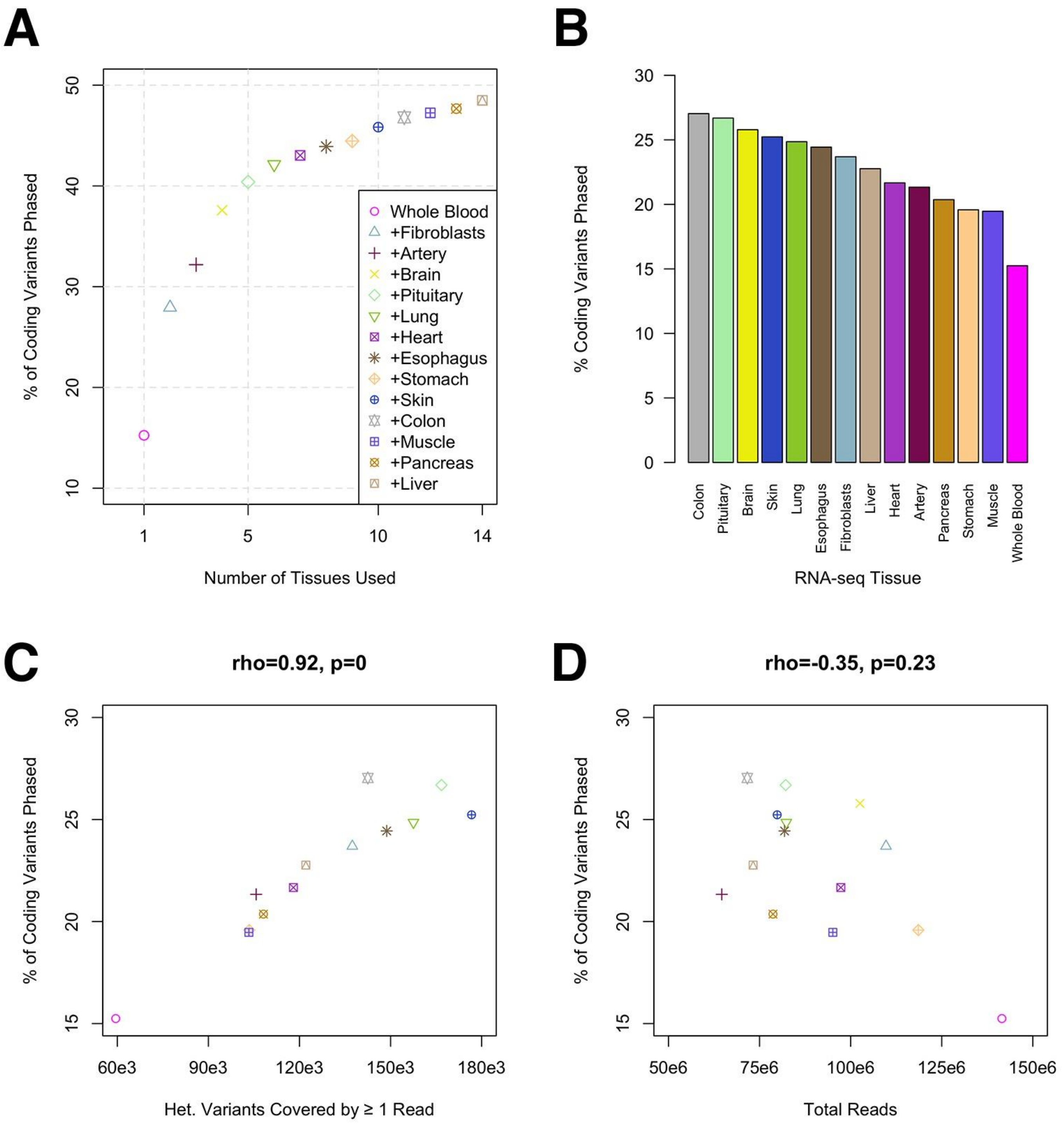

Supplementary Figure 5. Joint RNA-seq based phasing over multiple tissues greatly improves the number of variants that can be phased in an individual. A) Percentage of coding variants that can be phased beginning with RNA-seq from whole blood, and progressively adding data from up to 14 other distinct tissues from GTEx individual ZAB4 [12]. B) Percentage of coding variants that can be phased using each tissue from (A) individually. C-D) Percentage of coding variants as a function of the number of heterozygous variants covered by at least one read (C) or total library reads (D) for each tissue from (A) individually.

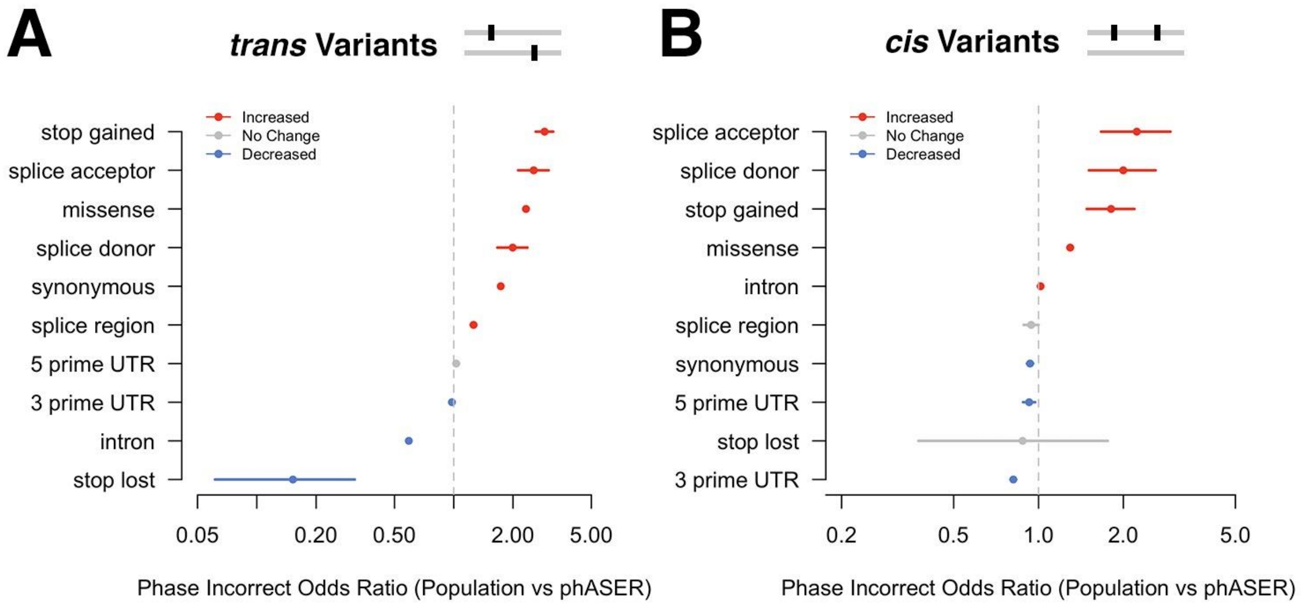

Supplementary Figure 6. Read backed phasing improves the ability to correctly identify instances of compound heterozygosity at rare variants. Cases of compound heterozygosity were called in 345 individuals using either 1000 Genomes Phase 3 population phasing or exome [15] + Geuvadis LCL RNA-seq [14] read backed phasing with phASER. The odds ratio for cases involving each variant type being incorrect in population data versus other types in either *trans* (A) or *cis* (B) interactions was calculated using Fisher's exact test. Variant types with a significantly increased probability of being incorrect when population phasing is used are shown in red, while those with a decrease are shown in blue ($p < 0.05$). Error bars indicate the 95% confidence interval of the odds ratio.

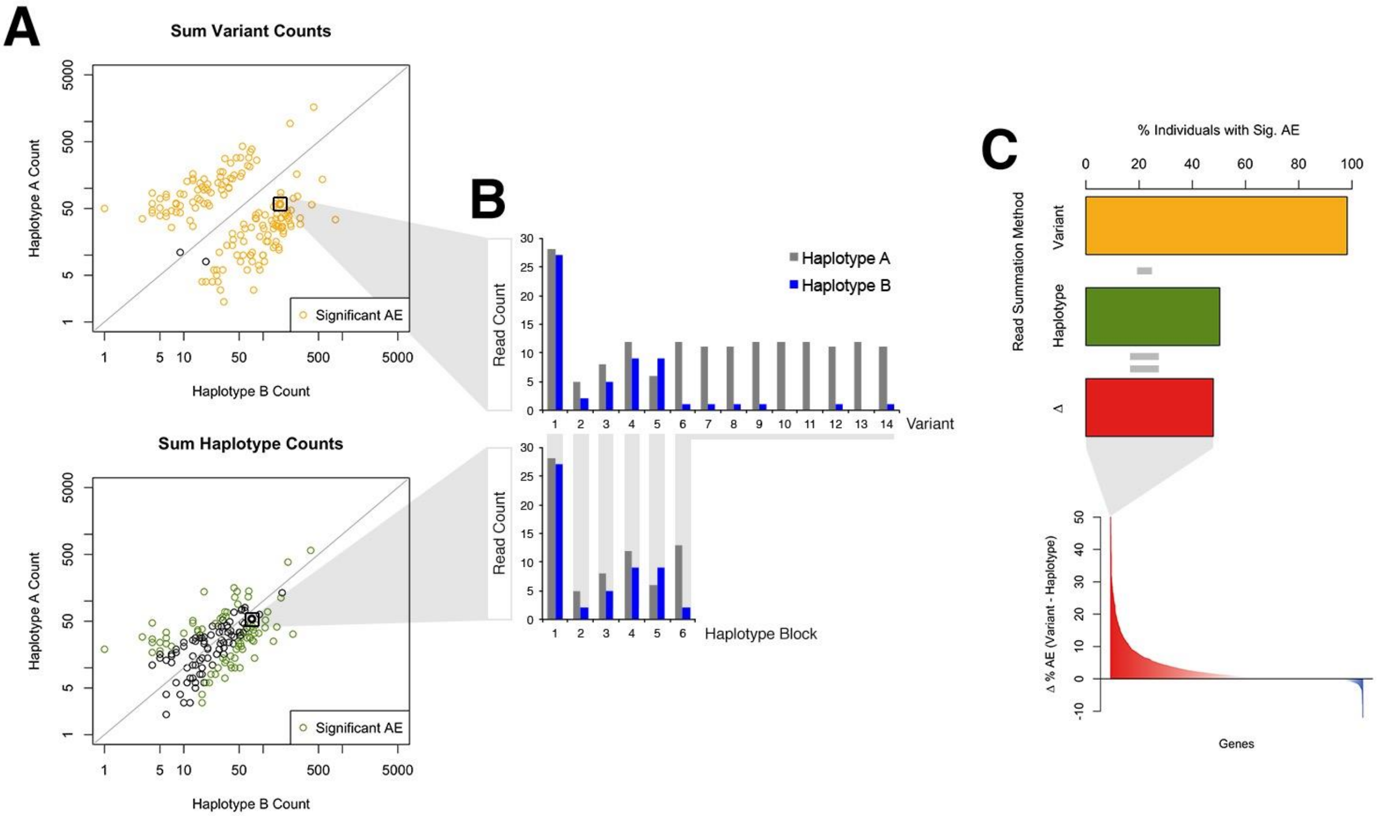

Supplementary Figure 7. Integrating allelic counts over variants using accurate phasing reduces false positives in allelic expression studies. A) Haplotypic counts for Geuvadis individuals at an example gene (ENSG00000162654 or *GBP4*) calculated by either summing counts from individual variants using 1000 Genomes Phase 3 population phasing [15] (top plot, yellow) or with phASER haplotypic counts (bottom plot, green). Each point represents one of the 345 individuals used in the analysis. B) Example illustrating that summing variant counts for the individual highlighted in (A) leads to double counting of variants 7 through 14 in this haplotype (top plot) and is prevented when haplotypic counts are used (bottom plot). C) For this gene the difference (red) in the percentage of individuals showing significant (5% FDR, binomial test) allelic imbalance calculated either by either summing variants (yellow) or using haplotypic counts (green). This value was calculated for all genes with allelic expression data from at least 30 individuals heterozygous for the top Geuvadis *cis*-eQTL (bottom plot and Fig. 2c).
